# Which inflammatory marker, between systemic immune-inflammation index and neutrophil to eosinophil ratio, is associated with Peyronie’s disease and are there any implications for a better understanding of its mechanisms?

**DOI:** 10.1186/s12610-023-00213-y

**Published:** 2023-12-19

**Authors:** Felice Crocetto, Ciro Imbimbo, Biagio Barone, Davide Turchino, Umberto Marcello Bracale, Antonio Peluso, Marco Panagrosso, Alfonso Falcone, Benito Fabio Mirto, Luigi De Luca, Enrico Sicignano, Francesco Del Giudice, Gian Maria Busetto, Giuseppe Lucarelli, Gaetano Giampaglia, Celeste Manfredi, Matteo Ferro, Giovanni Tarantino

**Affiliations:** 1 Department of Neurosciences, Reproductive Sciences and Odontostomatology, University of Naples “Federico II”, 80131 Naples, Italy; 2 Division of Urology, Department of Surgical Sciences, AORN Sant’Anna e San Sebastiano, 81100 Caserta, Italy; 3 Department of Public Health, Vascular Surgery Unit, University of Naples Federico II, 80131 Naples, Italy; 4 Division of Urology, AORN “Antonio Cardarelli”, Naples, Italy; 5 Department of Maternal Infant and Urologic Sciences, Policlinico Umberto I Hospital, “Sapienza” University of Rome, 00161 Rome, Italy; 6 Department of Urology and Organ Transplantation, University of Foggia, 71122 Foggia, Italy; 7 Urology, Andrology and Kidney Transplantation Unit, Department of Emergency and Organ Transplantation, University of Bari, 70124 Bari, Italy; 8 Department of Urology, IEO, European Institute of Oncology IRCCS, 20141 Milan, Italy; 9 Department of Clinical Medicine and Surgery, Federico II Medical School of Naples, Naples, Italy

**Keywords:** Peyronie’s disease, Neutrophil-to-eosinophil ratio, Eosinophil to neutrophil ratio, Systemic immune-inflammation index, Immuno-inflammatory response

## Abstract

**Background:**

Peyronie’s disease affects up to 9% of men and is often accompanied by pain and/or erectile dysfunction. It is characterized by an inflammatory process that is the grassroots of the subsequent fibrosis stage. There is an unmet need to evaluate its onset and progression. Among the newly proposed biomarkers of inflammation, authors developed a novel systemic immune-inflammation index (SII) based on lymphocyte, neutrophil, and platelet counts. Similarly, a recent study reported that a neutrophil-to-eosinophil ratio (NER) represents systemic inflammation.

**Results:**

A 49-patient group with Peyronie’s disease as confronted with 50 well-matched for age and BMI controls. As laboratory evaluation of inflammation, SII, NER and the eosinophil to neutrophil ratio (ENR) were studied. As a likely risk factor for the presence of Peyronie’s disease, a higher prevalence of hypercholesterolemia, hyperglycemia and hypertension was discovered in the patients compared to controls. A significant difference was found in the median values of the NER between the two selected groups, i.e., 32.5 versus 17.3 (*p* = 0.0021). As expected, also ENR was significantly different. The receiver operating characteristic curves for SII, ENR and NER were 0.55, 0.32 and 0.67, respectively, highlighting the best performance of NER. The cut-off for NER was 12.1, according to the Youden test.

**Conclusions:**

According to our results, any evaluation of circulating eosinophil, evaluated as NER, beyond being a signature of immuno-inflammatory response, help assess tissue homeostasis, since eosinophils are now considered multifunctional leukocytes and give a picture of the inflammatory process and repair process belonging to Peyronie’s disease.

## Résumé

**Contexte:**

La maladie de La Peyronie touche jusqu’à 9% des hommes et s’accompagne souvent de douleurs et/ou de dysfonction érectile. Elle se caractérise par un processus inflammatoire qui est. à la base de l’étape de fibrose ultérieure. Il existe un besoin non satisfait d’en évaluer son apparition et sa progression. Parmi les biomarqueurs de l’inflammation nouvellement proposés, les auteurs ont développé un nouvel indice d’inflammation immunitaire systémique (SII) basé sur le nombre de lymphocytes, de neutrophiles et de plaquettes. De même, une étude récente a rapporté qu’un rapport neutrophiles/éosinophiles (NER) représente une inflammation systémique.

**Résultats:**

Un groupe de 49 patients atteints de la maladie de La Peyronie a été confronté à 50 témoins étroitement appariés sur l’âge et l’IMC. Dans le cadre de l’évaluation de l’inflammation au laboratoire, le SII, le NER et le rapport éosinophiles/neutrophiles (ENR) ont été étudiés. En tant que facteur de risque probable de la présence de la maladie de La Peyronie, une prévalence plus élevée d’hypercholestérolémie, d’hyperglycémie et d’hypertension a été découverte chez les patients par rapport aux témoins. Une différence significative a été constatée pour les valeurs médianes du NER entre les deux groupes sélectionnés, c’est-à-dire 32,5 contre 17,3 (*p* = 0,0021). Comme on pouvait s’y attendre, le ERN était également significativement différent. Les courbes caractéristiques de fonctionnement du récepteur pour le SII, l’ENR et le NER étaient respectivement de 0,55, 0,32 et 0,67, ce qui met en évidence les meilleures performances du NER. Le seuil pour le NER était de 12,1 (test de Youden).

**Conclusions:**

D’après nos résultats, toute évaluation de l’éosinophilie circulante, sous la forme NER, au-delà d’être une signature de la réponse immuno-inflammatoire, permet d’évaluer l’homéostasie tissulaire, puisque les éosinophiles sont maintenant considérés comme étant des leucocytes multifonctionnels, et donne une image du processus inflammatoire et du processus de réparation appartenant à la maladie de La Peyronie.

**Mots-clés:**

Maladie de La Peyronie Rapport Neutrophiles/Eosinophiles Rapport Eosinophiles/Neutrophiles Indice d’Inflammation immunitaire systémique Réponse Immuno-inflammatoire

## Introduction

The aetiology of Peyronie’s disease (PD) is not completely known. Defined as a fibrotic disease, the evolution of pathophysiological knowledge in recent years as well as new studies seems to be related to penile trauma as one of the main causes of the disease. Indeed, penile trauma provokes a delamination of the tunica albuginea with a consequent small hematoma which successively progresses as inflammation and the subsequent accumulation of inflammatory cells and production of reactive oxygen species (ROS). In the course of the inflammation, PD develops due to the activation of nuclear factor kappa-B, which causes the production of inducible nitric oxide synthase and a consequent increase of nitric oxide, leading to augmented generation of peroxynitrite anion [1]. As result, repetitive microvascular injury and fibrin deposition not adequately cleared during the normal remodeling and repair of tunica, leads to fibroblasts activation and proliferation in addition to enhancing vessel permeability and generation of chemotactic factors for leukocytes [2]. Fibrin act indeed as a strong chemoattractant, promoting the inflow of inflammatory cells such as macrophages, neutrophils, mast cells, cytokines, and fibroblasts [3]. Various pro-inflammatory cytokines such as transforming growth factor beta-1 and platelet-derived growth factor are released by these inflammatory cells [4]. Another key-pathogenetic mechanism consists of an impairment of the so-called endothelium-dependent flow-mediated dilation (FMD) that has been found in PD patients compared to controls [5]. Endothelial dysfunction is a condition of impaired endothelium-dependent vasodilation and, most important, of endothelial activation, characterized by a pro-inflammatory, proliferative, and pro-coagulatory milieu [6]. Platelet adhesion to endothelium and the process of leukocyte rolling on the same layer represents the first step of a transition-state leading to diapedesis, platelet-leukocyte interaction and finally aggregation on a thrombogenic surface and vascular occlusion [7]. Among the newly proposed biomarkers of inflammation, several authors have utilized a novel systemic immune-inflammation index (SII) based on lymphocyte, neutrophil and platelet counts, exploring its prognostic value in various cancers and diseases, with the rationale that cancer and inflammation share a common microenvironment [8–14]. Furthermore, SII had a better prediction of major cardiovascular events than traditional risk factors in coronary artery disease patients after coronary intervention [15]. Another novel biomarker represented by neutrophil-to-eosinophil ratio (NER) has been associated with higher odds of in-hospital mortality in acute ischemic stroke patients as well as with in-hospital mortality of patients with chronic obstructive pulmonary disease [16, 17]. Conversely, considering the role of inflammation in carcinogenesis, a lower baseline NER was associated with improved clinical outcomes in patients with metastatic renal cell carcinoma treated with nivolumab plus ipilimumab as well as with improved clinical outcomes in patients treated with pembrolizumab for advanced urothelial cancer [18, 19]. Nevertheless, according to some interpretations, limited by the accuracy of the instrument, eosinophil count may show a number of zero in some patients thus excluding these patients from the ratio and introducing a potential bias. Therefore, for some authors eosinophil-to-neutrophil ratio (ENR) may be a more stable biomarker than NER [20]. The importance of finding a simple and reliable inflammatory marker to weigh the presence or the course of PD is testified by a recent piece of research demonstrating that neutrophil-to-lymphocyte ratio (NLR) could be helpful to differentiate the chronic phase from the acute phase in patients with PD. Authors concluded that NLR could be used as an objective biomarker for the management of the disease and for choosing the appropriate treatment [21]. The aim of the present study was to select which inflammatory marker among NER, ENR and SII, all of them representing simple and feasible markers that are easily calculated in the complete blood count, was associated with PD, in particular in the phase in which the inflammatory process influences connective tissue and leads to fibrotic alterations in the tunica albuginea [22].

## Material and methods

### Study design

In this observational retrospective study, we utilized the same patient sample included in a previous research according to The International Committee of Medical Journal Editors (IC-MJE) [23, 24]. This study is in compliance with the ethical guidelines of the Declaration of Helsinki (1975). Written informed consent was obtained before proceeding with the study by all patients involved. The ethical committee of the Federico II University Medical School of Naples gave its approval.

### Inclusion criteria

Forty-nine male patients were enrolled in this study, fulfilling the diagnostic criteria of stable PD. Fifty male individuals without PD, well-matched for age and BMI, admitted to our department with the diagnosis of benign prostatic hyperplasia (n 23) or undergone extracorporeal shock wave lithotripsy for the treatment of renal-ureteral lithiasis (n 27) were selected as a control group. PD patients and Controls who had different grades of obesity were on a calorie-reduced, low-fat diet. If suffering from co-morbidities, such as type 2 diabetes mellitus and hypertension, they were on drugs obtaining metabolic and hemodynamic control.

The inclusion criteria for patient enrolment were as follows: (a) the presence of PD; (b) the availability of complete peripheral blood counts.

The accuracy of all clinical, laboratory and imaging data obtained from the institutional databases was validated for each patient by an independent observer using the medical records. Data was collected into electronic data files by the local urologists and opportunely checked at the central data management.

### Exclusion criteria

Patients were carried out if they had a decrease in weight loss in the past months (i.e., 10% initial body weight, due to hidden cancer) or recent acute illness (viral, fungal or bacterial infection) that might have influenced laboratory inflammatory parameters.

Patients presenting with suspicion or evidence of hematological system diseases, chronic inflammatory diseases such as ankylosing spondylitis, psoriasis, inflammatory bowel disease and the use of anti-inflammatory medicines were ruled out. Similarly, men suffering from major cardiovascular diseases, both previous or in course, were disallowed.

### Diagnostic criteria for Peyronie’s disease

First of all, medical history from patients with suspected PD (based on physical examination) was collected. Subsequently, patients underwent dynamic penile color-doppler ultrasound scan (US), in order to evaluate penile curvature and the ultrasonographic appearance of tunica albuginea. Finally, International Index of Erectile Function-5 questionnaire, consisting of 5 items, was submitted to the patients.

### Alcohol consumption

Enrolled individuals were categorized as non-drinkers or moderate drinkers if they have limited intake to 2 drinks or less in a day according to Dietary Guidelines for Americans 2020–202 5 [25].

### Exercise

Patients were categorized as having a sedentary lifestyle or doing at least 150 minutes of moderate-intensity aerobic physical activity throughout the week or at least 75 minutes of vigorous-intensity aerobic physical activity throughout the week or an equivalent combination of moderate- and vigorous-intensity activity, according to WHO guidelines on physical activity and sedentary behavior [26].

### Ultrasonography features

As previously mentioned, the diagnosis of PD was made by an experienced urologist via physical examination, performed examining the penis in order to identify a palpable penile plaque in flaccid and stretched state. Successively, the US analysis was carried out utilizing an ultrasound scan equipped with a 7-12 MHz multi-frequency linear probe (BK Flex Focus 800, BK medical System Inc., United States), with the patient in the supine position. B-mode US study was performed in transversal and longitudinal planes starting at the level of the glans and moving down to the base of the penis, with the penis placed toward the abdomen and the transducer placed at the ventral surface of the penis. 10 micrograms of prostaglandin E1 were injected into the left corpus cavernosum via a 25-gauge insulin injector in order to perform the doppler study and evaluate the presence of non-palpable plaques with erected penis. In particular, calcified penile plaques were detected as focal hyperechoic thickening of the tunica albuginea with 6 of the acoustic beam while non-calcified plaques were isoechoic or slightly hyperechoic compared with the surrounding tunica albuginea. The imaging features of the PD patients were presented elsewhere [23].

### Anthropometric evaluation

Normal weight was considered as body mass index (BMI) between 18.5 and 24.9, overweight a BMI between 25 and 29.9, while obesity was characterized by a BMI of 30 or more.

### Metabolic profile

Type 2 diabetes mellitus (T2DM) was diagnosed in the presence of fasting plasma glucose concentrations ≥126 mg dL-1, or on antidiabetic agents.

### Laboratory evaluation of inflammation

Blood samples were collected from all participants in the early morning after an overnight fast. An automated hematologic analyzer (Coulter LH750) was used to measure total and differential blood parameters/counts. The SII was calculated as follows: SII = P × N/L, where P, N, and L were the peripheral blood platelet, neutrophil, and lymphocyte count (number of cells × 10^3^/μL) according to Lolli et al. [27]. The NER was calculated by the absolute neutrophil count (number of cells × 10^3^/μL) divided by absolute eosinophil count (number of cells × 10^3^/μL), according to Tucker et al. [18]. ENR was obtained by inverting the factors, i.e., eosinophil count divided by neutrophil count.

### **Further** l**aboratory assessment**

Fasting plasma glucose (n.v. 70–100 mg/dL), lipid profile comprehending serum levels of triglycerides (TG) (n.v. < 150 mg/dL), total cholesterol (TC, n.v. < 200 mg/dL), high density lipoprotein (HDL) cholesterol, n.v. > 40 mg/dL, low density lipoprotein (LDL) cholesterol, n.v. < 100 mg/dL, international normalised ratio (INR, n.v. 0.9–1-1) and serum creatinine (n.v. 0.72–1.25 mg/dL) were measured according to in-house procedures.

### Statistics

Data derived from a normally distributed population was presented as mean plus SD. Variables not normally distributed or ordinals were expressed as median (25–75 IQR). The difference in medians was assessed by the Wilcoxon rank-sum test (Mann-Whitney test), while for evaluating the difference between means, the independent t-test was used. For assessing frequencies we applied a two-way table with measures of association, calculating the Fisher’s exact test that is more accurate than the chi-square test or *G*–test of independence when the expected numbers are small [28]. The extended Mantel-Haenszel with ANOVA (transformation in ranks) analysis, also called the Friedman test, was used when NER values were adjusted for some frequencies [29]. Logistic regression was used to predict the presence/absence of a dependent variable, i.e., PD, by both SII and NER or ENR (variables found significantly different in the two groups) reporting Odds ratio, Std. err., t, P > |z| and 95% CI. The R-square statistic was used for assessing the predictive strength of the logistic regression model. The collinearity was assessed in the presence of a value of the variance inflation factor (VIF) superior to 2.5 and a value of the tolerance inferior to 0.10. As evident, for appreciating VIF a more conservative size was applied.

ROC analysis (DeLong method) was used as a diagnostic decision making between the groups (patients with and without PD). Indicatively, to measure the performance of the binary classification test (index test), the area under the receiver operating characteristic (AUROC/AUC) was performed to evaluate the most appropriate models (the highest specificity and sensitivity), under the nonparametric assumption. The correct classification with related sensitivity and specificity was performed using the Probit model. The test equality of more ROC areas was performed to compare the performance of several variables. The best cut-off, coupled with the sensitivity, specificity, positive likelihood ratio, and negative likelihood, was studied [30]. The cut-off with the highest specificity and sensitivity was calculated by means of the Youden Index according to Fluss et al. [31]. Stata 17.0 (Copyright 1985–2021, 4905 Lakeway Drive, College Station, Texas 7784) was used for statistics.

## Results

**Fig. 1:**
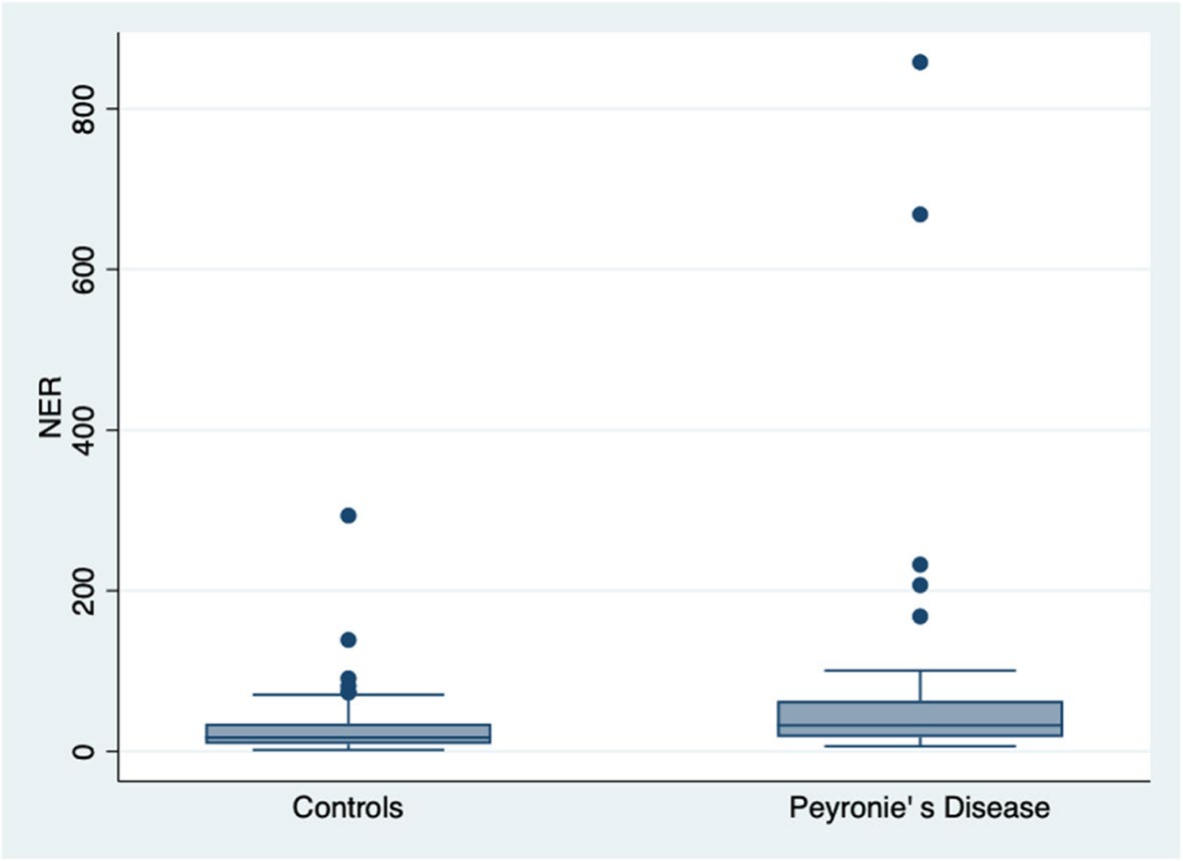
The behaviour of NER in patients with Peyronie’s disease and controls

The main characteristics of the whole population are shown in Table 1. The lack of significant differences concerning age, BMI and marital status showed that the two groups were well-matched. The prevalence of T2DM between the two groups was similar, while the prevalence of hypertension was greater in the PD group (*p* = 0.017) as well as of hypercholesterolemia (*p* = 0.0001). There were significant differences in the median values of total cholesterolemia and plasma glucose between the PD group and controls (Table 1). The median of INR was different between the two cohorts but was comprised in the normal range. The most relevant result was the significant difference in the median values of the NER between the group of PD patients and the controls (32.5 versus 17. 3, *p* = 0.0021, Wilcoxon rank-sum test), with limited overlapping (Fig. 1) (Table 2). This difference was maintained also when adjusting for hypertension and hypercholesterolemia i.e., using extended Mantel-Haenszel (Cochran-Mantel-Haenszel) Stratified Test of Association, Q (1) = 7.18, *p* = 0.0074 and 7.22, *p* = 0.0072, respectively. Most interestingly, the previous difference concerning NER, when adjusted for the values of plasma glucose, was lost, i.e., Q (1) = 1.43, *p* = 0.23, independently from the prevalence of T2DM that was similar. As expected, ENR mirrored the same behavior of NER. Vice versa the SII did not show any difference in its median values between the two selected populations. There was a trend in the difference of median values of HDL-cholesterol between the two selected cohorts, but not concerning LDL-cholesterol. Physical activity was found of overlapping intensity between the PD patients and the controls (*p* = 0.70).

**Table 1:** Clinical and laboratory data of the two groups

Variables	Peyronie’s disease n 49	Controls n 50	p
**Age (years, mean ± SD)** *a*	61.4 ± 9.8	60.2 ± 8.1	0.48
**BMI (mean ± SD)** *a*	26.4 ± 3	25.6 ± 2.3	0.15
**Normoweight/Overweight/Obese (n)** *c*	15/27/7	20/28/2	0.17
**T2DM (yes/no, n)** *c*	7/42	3/47	0.18
**Hypertension (yes/no, n)** *c*	28/21	17/33	0.017
**Alcohol consumption (yes/no, n)** *c*	12/37	16/34	0.50
**Exercise** **(yes/no, n)** *c*	29/20	20/30	0.07
**Fasting Plasma Glucose (mg/mL, median + IQR)** *b*	101 (85–115)	87 (77–98)	0.0088
**Hyper-cholesterolemia (> 200 mg/dL, n)** *c*	25/24	7/43	0.0001
**Total Cholesterol (mg/dL, median + IQR)** *b*	187 (162–203)	159 (136–192)	0.0018
**HDL-cholesterol (mg/dL, median + IQR)** *b*	45 (35–59)	40.5 (32–51)	0.053
**LDL-cholesterol (mg/dL, median + IQR)** *b*	95 (85–131)	89.7 (61.8–131.6)	0.11
**Creatinine (mg/dL, median + IQR) b**	0.9 (0.8–1.3)	0.975 (0.82–1.16)	0.08
**INR (median + IQR)** *b*	0.94 (0.92–0.95)	1.01 (0.97–1.08)	0.0001
**SII** *b*	529 (404.5–846.7)	498.4 (296.5–776.8)	0.38
**NER** *b*	32.5 (18–62.8)	17.3 (9.38–34.5)	0.0021
**ENR** *b*	0.030 (0.016–0.055)	0.036 (0.03–0.10)	0.0021

*BMI* body mass index, *ENR* eosinophil to neutrophil ratio, *HDL* high density lipoprotein, *INR* International Normalized Ratio, *IQR* interquartile range, *LDL* low density lipoprotein, *n* number, *NER* neutrophil to eosinophil ratio, *p* significance, *SD* standard deviation, *SII* systemic immune-inflammation index, *T*2*DM* type 2 diabetes mellitus, The symbol *a* indicates that the for evaluating the difference between means was used the independent, while difference in medians was assessed by the Wilcoxon rank-sum test (Mann-Whitney test), and was indicated by the symbol *b*. The two-way table with measures of association, calculating the Fisher’s exact, was indicate with the symbol *c*

**Table 2:** Behavior of the main inflammatory parameters of the two selected groups

	SII systemic immune-inflammation index		
IQR	Peyronie’s disease	Controls	***p***
25%	404.5	296.57	0.38
50%	529	498.39	0.38
75%	846.7	776.88	0.38
	**Neutrophil-to-eosinophil ratio**		
25%	18	9.38	0.0021
50%	32.51	17.31	0.0021
75%	62.87	34.5	0.0021
	**Eosinophil to neutrophil ratio**		
25%	0.15	0.28	0.0021
50%	0.30	0.57	0.0021
75%	0.55	0.106	0.0021

*IQR* Interquartile range.; p, significance. The difference in medians was assessed by the Wilcoxon rank-sum test (Mann-Whitney test)

### Predictions

Both the NER and ENR well predicted the presence of PD at logistic regression analysis, without any collinearity, as evident in Table 3. There was no collinearity among the examined variables as evidenced by a mean value of VIF largely inferior to 2.5. The R-square of the model was relatively low (R-square = 0.1178), indicating a moderate predicting power. To clarify this last aspect, ROCs were carried out.

**Table 3:** Prediction of PD by the values of NER, ENR and SII

	Coefficient	STD err	t	P	VIF
NER	0.00060	0.00024	2.44	0.017	1.14
ENR	−1.97280	0.55866	−3.53	0.001	1.13
SII	−0.00007	0.00004	−1.76	0.082	1.11

*ENR* eosinophil to neutrophil ratio, *NER* neutrophil to eosinophil ratio, *SII* systemic immune-inflammation index, *STD err* standard error, *VIF* inflation factor. The absence of collinearity in the logistic regression model was assessed by a value of VIF inferior to 2.5 and a value of the tolerance superior to 0.10. The R-square of 0.1178 showed a moderate predicting power

**Fig. 2:**
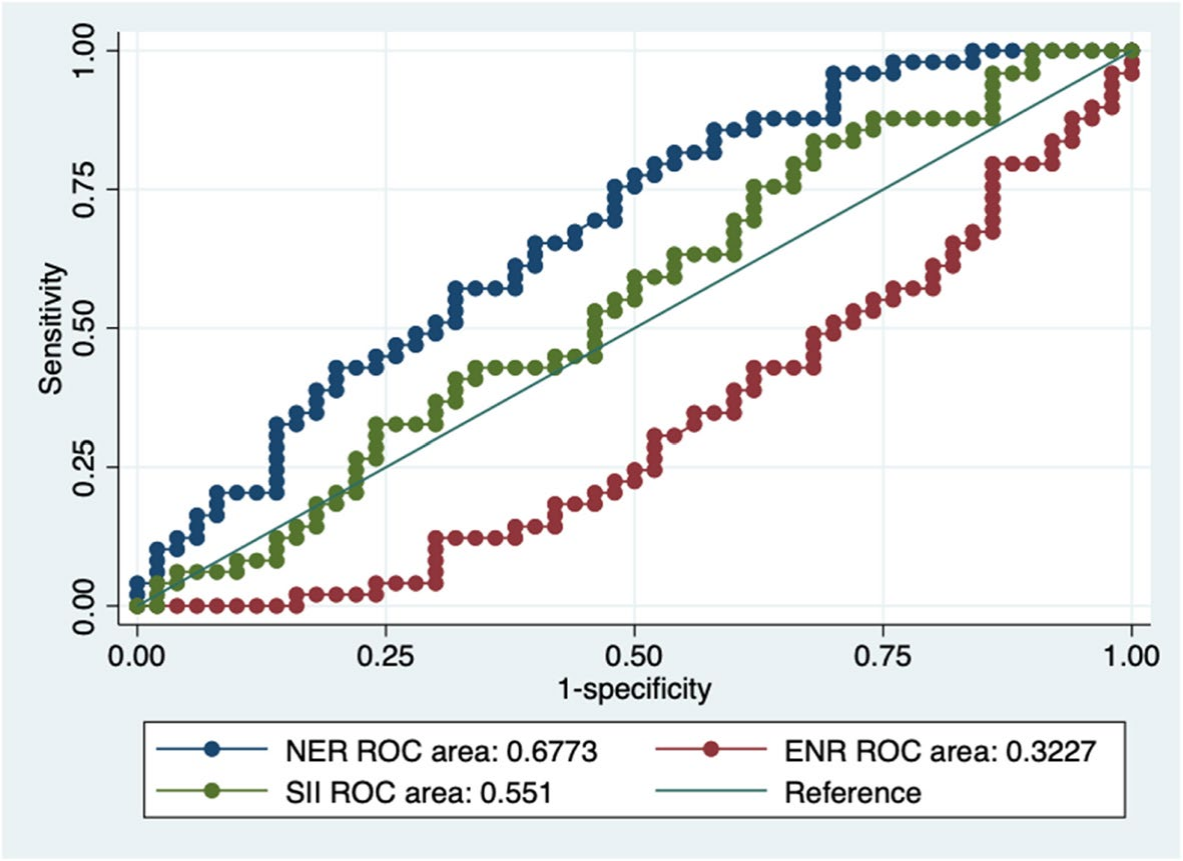
The AUROCs of NER, ENR and SII in diagnosing Peyronie’s disease

The ROC analysis on 99 observations, used as a diagnostic decision making between the groups (patients with and without PD), among NER, ENR and SII, showed that NER best performed, i.e., AUC = 0.6773 (95% C. I 0.572–0.7826). SII and ENR ROCs were AUC = 0.551 and AUC = 0.3227, respectively (Fig. 2).

### Sensitivity/specificity analysis

The correct classification with related sensitivity and specificity was performed using the Probit model, showing an overall rate of correct diagnosis of 58.59%. Specifically, 82% of the control group and 44.69 of PD group were correctly classified by NER. The best cut-off of NER was obtained by calculating the Youden Index, resulting to be 12.10, which yielded a sensitivity of 0.755 and a specificity of 0.50.

## Discussion

PD is characterized by post-inflammatory fibrotic plaques in the penile tunica albuginea that cause curvature of the erect penis and affects up to 9% of men. It is often accompanied by pain and/or erectile dysfunction in addition to disruption of psychological and emotional aspects linked to sexuality [22]. The overall prevalence of emotional and relationship problems attributable to PD was, indeed, 81 and 54%, respectively. Risk factors include age and marital status but also the duration and the stability of disease play an important role [32]. Therefore, trying to assess the inflammatory processes that cause the onset of the disease is of paramount importance in the early assessment of PD. Similarly, considering the impact of the molecular initiation events on its stability and duration, there is an unmet need to target this aspect to lessen the related psychological difficulties. Consistent with our results, the inflammatory marker NER is closely associated with PD and could be useful to confirm PD in the first stages and eventually in the follow-up of the disease.

As previously emphasized, apart from the impairment of the FMD, another key-pathogenetic mechanism of PD consists in fibrosis [5]. It is noteworthy that ureteral strictures, retroperitoneal fibrosis and PD are characterized by extracellular matrix abnormalities, such as collagen deposition, transforming growth factor-β accumulation, and dysregulation of collagen maturation-leading to abnormal tissue stiffness. Consequently, they share a systemic pro-inflammatory state likely contributing to their associated fibrogenesis [33]. To our knowledge, this is the first study to explore the role of NER and ENR as a potential biomarker associated with PD. Indeed, only other two similar studies, analyzing the role of inflammatory parameters associated with PD have been published. Ozbir et al., in 2019, analyzed the role of neutrophil-to-lymphocyte ratio (NLR), platelet-to-lymphocyte ratio (PLR) and monocyte-to-eosinophil ratio (MER) in discriminating the phases of PD. The authors reported a statistically significant difference in NRL and PLR among patients with acute and chronic disease with the first that was statistically significant also at a multivariate regression analysis [21]. Analogously, Garcia Rojo et al., more recently, evaluated the role of NLR and PLR in PD in relation to acute and chronic phases of PD, reporting a statistically significant difference for both ratios in discriminating the phase of the disease. Differently from the previously reported studies [34] the aim of our work was to evaluate the role of inflammatory biomarkers in PD patients versus no PD controls, using patients with stable PD. Currently, no specific blood tests are available for the diagnosis of PD. The possibility to utilize a simple, quick and inexpensive blood count could represent a further way towards the early diagnosis and comprehension of PD. We try to posit a hypothesis as to why inflammatory markers based on eosinophil count could partially clarify some mechanisms of PD, specifically the regenerative process. Many clinical and pre-clinical models have shown that eosinophils play a vital role in both the immunological response (type 2) and inflammatory process. The type 2 immune response is crucial for tissue repair and, during this phase, eosinophils play crucial roles in regeneration [35, 36]. Eosinophils are recruited from bone marrow and blood to the sites of immune-inflammatory response. Among various hematopoietic factors, interleukin (IL)-3, granulocyte-macrophage colony-stimulating factor (GM-CSF), and mainly IL-5 are central to eosinophil proliferation and differentiation, even though IL-3 and GM-CSF also stimulate proliferation of neutrophils and basophils [37]. What is more, the regenerative process is of a paramount importance in PD because the development of scar involves the deposition of connective tissue. Accordingly, recent results show that eosinophils contain a metalloprotein that degrades types I and III collagens [38].

Summarizing up, any evaluation of circulating eosinophil, beyond being a signature of immuno-inflammatory response, helps assess tissue homeostasis, due to the fact that eosinophils are now considered multifunctional leukocytes [39]. The finding that SII does not bear any association with PD can be explained by the fact that this marker is composed of other types of leucocytes but eosinophils, independently from the fact that PD is prevalently characterized by a local inflammation without any systemic involvement.

### Limitations of the study

This work provides further evidence that baseline eosinophil count, evaluated as NER, mirrors scarce recruitment of this type of leukocyte in the place of inflammation. Our study has different limitations, which include the retrospective and descriptive nature of the study in addition to the partially reduced sample size.

## Conclusion

According to our results, blood hypertension, beyond hypercholesterolemia and moderate hyperglycemia, may be considered statically significant risk factors for developing PD as previously demonstrated [40].

### Future directions

Potential drugs may lead to improved response to inflammation, via eosinophil stimulation, such as IFN gamma [41]. Further studies are required in order to evaluate the impact of the routine use of NER in clinical practice and to confirm its potential role in the diagnostic pathway of PD.

## Data Availability

The raw data supporting the conclusions of this article will be made available by the authors, without undue reservation.

## Abbreviations

PD: Peyronie’s disease
ROS: Reactive oxygen species
FMD: endothelium-dependent flow-mediated dilation
NER: Neutrophil-to-eosinophil ratio
ENR: Eosinophil-to-neutrophil ratio
NLR: Neutrophil-to-lymphocyte ratio
US: Ultrasound scan
BMI: Body mass index
T2DM: Type 2 diabetes mellitus
TG: Triglycerides
HDL: High density lipoprotein
LDL: Low density lipoprotein
VIF: Variance inflation factor
ROC: Receiver operating characteristic
GM-CSF: Colony-stimulating factor
